# Identification of the *C. elegans *anaphase promoting complex subunit Cdc26 by phenotypic profiling and functional rescue in yeast

**DOI:** 10.1186/1471-213X-7-19

**Published:** 2007-03-20

**Authors:** Yan Dong, Aliona Bogdanova, Bianca Habermann, Wolfgang Zachariae, Julie Ahringer

**Affiliations:** 1 The Gurdon Institute and Department of Genetics, University of Cambridge, Tennis Court Road, Cambridge CB2 1QN, UK; 2 Max Planck Institute of Molecular Cell Biology and Genetics, Pfotenhauerstrasse 108, 01307 Dresden, Germany

## Abstract

**Background:**

RNA interference coupled with videorecording of *C. elegans *embryos is a powerful method for identifying genes involved in cell division processes. Here we present a functional analysis of the gene B0511.9, previously identified as a candidate cell polarity gene in an RNAi videorecording screen of chromosome I embryonic lethal genes.

**Results:**

Whereas weak RNAi inhibition of B0511.9 causes embryonic cell polarity defects, strong inhibition causes embryos to arrest in metaphase of meiosis I. The range of defects induced by RNAi of B0511.9 is strikingly similar to those displayed by mutants of anaphase-promoting complex/cyclosome (APC/C) components. Although similarity searches did not reveal any obvious homologue of B0511.9 in the non-redundant protein database, we found that the N-terminus shares a conserved sequence pattern with the N-terminus of the small budding yeast APC/C subunit Cdc26 and its orthologues from a variety of other organisms. Furthermore, we show that B0511.9 robustly complements the temperature-sensitive growth defect of a yeast *cdc26Δ *mutant.

**Conclusion:**

These data demonstrate that B0511.9 encodes the *C. elegans *APC/C subunit CDC-26.

## Background

A major goal in biology is to understand the function of each gene. For many organisms, complete genome sequences are now available. Combined with knockdown techniques such as RNAi or morpholino oligos, genes can be quickly assayed for *in vivo *function [1,2]. In *C. elegans*, genome-wide knockdown of gene activity through RNAi has provided important phenotypic information for thousands of genes [3-8]. Although a useful starting point, much of the phenotypic information lacks detail; for example, many genes are only annotated as essential for viability.

Several studies have carried out additional analyses to identify more precise gene functions. In particular, RNAi videorecording screens have uncovered very detailed defects allowing genes to be grouped into more specific phenotypic classes [4,7-9]. However, within each class there still exist groups of genes with different functions. Analysing the phenotypes of individual genes in more depth is important for assigning genes to specific functions. Through an interest in embryonic polarity, we investigated the function of B0511.9, previously identified as having embryonic polarity defects in a large-scale RNAi videorecording screen [9]. Through phenotypic analyses, we show that B0511.9 shares functions with components of the cell cycle regulator, the anaphase promoting complex/cyclosome (APC/C).

The APC/C is a complex of 12 subunits in animal cells (13 in yeast) that regulates destruction of key cell cycle regulators at the appropriate times by targeting them for degradation by the 26 S proteasome through its E3 ubiquitin ligase activity (reviewed in [10-12]). In *C. elegans*, nine of the 12 APC/C subunits have been identified based on sequence analysis [13-17]; Cdc26, Apc7, and Apc13 were not identified. For seven subunits, loss of function using mutants or RNAi causes an arrest at metaphase of meiosis I; for two (*apc-5 *and *apc-10*), weak embryonic lethality was seen along with germline maintenance problems consistent with mitotic defects (reviewed in [18]).

## Results and Discussion

### B0511.9 is required for the metaphase to anaphase transition of meiosis I

In large scale RNAi videorecording screens, RNAi of B0511.9 was shown to cause different phenotypes: loss of asymmetry in the first cell division or one cell arrest due to failure to pass through meiosis [8,9]; To understand the role of B0511.9 in cell division, we examined the RNAi phenotype in detail.

We first carried out a time course of RNAi feeding of B0511.9 and examined embryos laid at different times after RNAi was initiated in the mother (Table 1). RNAi knockdown increases in strength during the time course. There was an increase in lethality from 17.9% at 24–32 hours post feeding to 100% at 56 hours post feeding or later. The terminal arrest phenotypes of the embryos changed over time. At 24–32 hours post feeding, most arrested embryos contained many cells whereas embryos laid 56 hours after initiation of RNAi feeding arrested as a single cell (Table 1). At intermediate time points, both types of terminal arrest embryos were seen (Table 1).

**Table 1:** Time course of RNAi of B0511.9

Time post RNAi	Dead embryos (n)	Hatched embryos (n)	Phenotype of dead embryos
24–32 hours	17.9% (93)	82.1% (425)	Multicellular
32–48 hours	31.1% (324)	68.9% (719)	One cell arrest or multicellular
48–56 hours	81.6% (288)	18.4% (65)	Predominantly one cell arrest
> 56 hours	100% (93)	0%	One cell arrest

**Figure 1:**
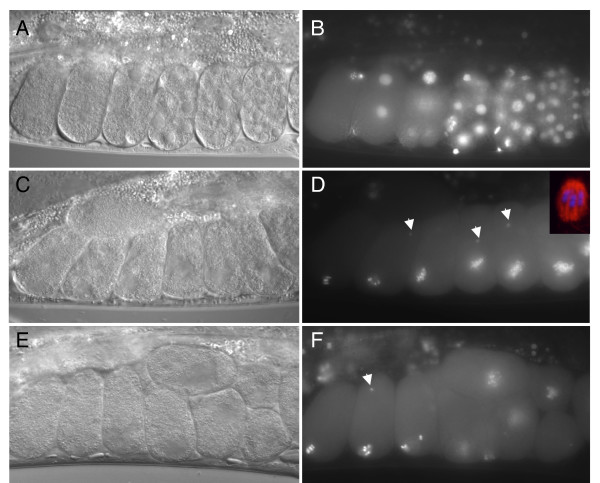
**Meiotic metaphase arrest induced by strong RNAi of B0511.9**. Pairs of pictures show DIC and fluorescence images of embryos in the uterus of a mother carrying a GFP::H2B transgene marking nuclei. (A, B) wild-type embryos show progressively more nuclei as divisions proceed. (C, D) *B0511.9(RNAi) *embryos are all arrested at the one cell stage; staining of such embryos for beta-tubulin (red) and DNA (blue) shows arrest stage is at meiotic metaphase I (inset). (E, F) *emb-27/Cdc16(RNAi) *embryos arrested in metaphase of meiosis I [15], a phenotype identical to that of *B0511.9(RNAi) *in (C, D). Arrows in (D) and (F) point to sperm chromatin, indicating that the embryos have been fertilized.

To investigate the arrest stage of the embryos after strong RNAi of B0511.9 we examined the pattern of DNA condensation of embryos inside the uterus using a GFP::histone reporter gene. Compared to the wild-type where progressively older embryos have progressively more cells (Figure 1A, B), embryos in the uteri of *B0511.9(RNAi) *mothers all showed the DNA condensation typical of metaphase of meiosis I (Figure 1C, D). Staining these arrested embryos for tubulin confirmed that embryos arrested with a meiosis I metaphase-like spindle (inset in Figure 1D). This phenotype is similar to that reported for mutants or strong RNAi of anaphase promoting complex/cyclosome (APC/C) subunits [13-15,17]. We confirmed that the phenotype of strong *B0511.9(RNAi) *embryos described above is identical to that seen after RNAi of APC/C component *emb-27/Cdc16 *(Figure 1E, F). These results indicate that like APC/C components, B0511.9 is required for progression from metaphase to anaphase of meiosis I.

### Weak depletion of B0511.9 causes embryonic polarity defects

We next examined the phenotype of embryos laid after shorter maternal RNAi of B0511.9, which should cause a weaker depletion. In wild-type embryos, the first cell division is asymmetric, occurring at 56% embryo length (range of 54–57%, n = 20). In contrast, we found that first division in *B0511.9(RNAi) *embryos is much more symmetric, occurring on average at 52% embryo length (range of 51–54%, n = 10). This suggests that *B0511.9(RNAi) *embryos have a defect in embryonic polarity.

In wild-type embryos, polarity is initiated during the first cell cycle, leading to the asymmetric localisation of PAR polarity proteins, with a complex of PAR-3/PAR-6/PKC-3 at the anterior cortex and PAR-1 and PAR-2 at the posterior cortex (reviewed in [19]). These proteins are required for the posterior displacement of the first mitotic spindle, which leads to an asymmetric cell division. In *par *mutant embryos, the first division is symmetric instead of asymmetric and the remaining PAR proteins are often mislocalized [19-26].

**Figure 2:**
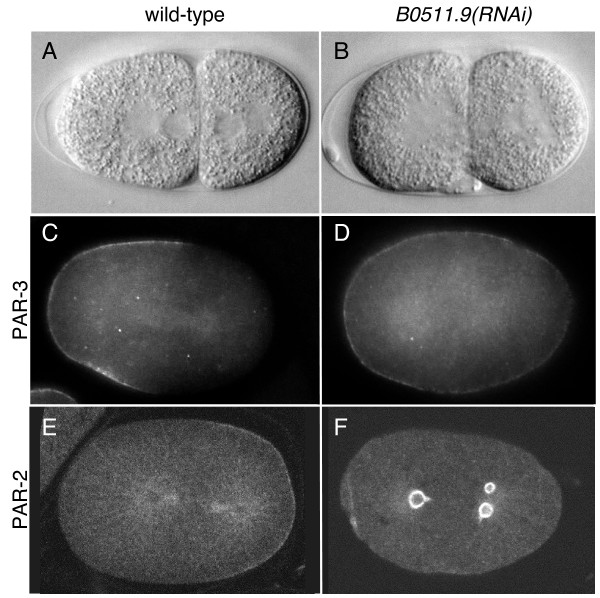
**Embryonic polarity defects after weak RNAi of B0511.9**. (A) wild-type 2 cell embryo after asymmetric first division; anterior AB cell is larger than the posterior P1 cell. (B) *B0511.9(RNAi) *embryo showing a symmetric first division. (C) PAR-3 at the anterior cortex of a wild-type one-celled embryo at anaphase. (D) PAR-3 on the entire cortex of a *B0511.9(RNAi) *embryo at anaphase. (E) PAR-2 at the posterior cortex of the wild-type one-celled embryo in (C). (F) PAR-2 in cytoplasmic structures in the *B0511.9(RNAi) *embryo shown in (D). The PAR-2 antibody shows weak cross-reaction with microtubules. PAR-2 and PAR-3 distributions were scored in wild-type and *B0511.9(RNAi) *embryos from prophase to the two cell stage. We distinguished weak versus strong classes of PAR staining defects in *B0511.9(RNAi) *embryos: (1) weak: an enlarged domain of cortical PAR-3 with a reduced domain of cortical PAR-2. (2) strong: complete cortical PAR-3 with PAR-2 in cytoplasmic puncta. In embryos where both meiotic divisions occurred, scored by the presence of two polar bodies, 50% were in the weak class and 17% in the strong class (n = 18). In embryos with a meiotic division defect, scored by the presence of only one polar body, 23% were in the weak class and 63% were in the strong class (n = 49). The PAR distribution defects in *B0511.9(RNAi) *embryos having two polar bodies suggests that its polarity function is separable from its meiotic function.

To determine whether RNAi of B0511.9 affected PAR polarity, we examined the localization of PAR-2 and PAR-3 in weakly affected *B0511.9(RNAi) *embryos. We found that these PAR proteins were abnormally distributed in 82% (n = 67) of such embryos, compared to 0% abnormal distribution in wild-type embryos (n = 30), indicating a defect in embryonic polarity. In most of B0511.9(RNAi) embryos, PAR-3 was expanded to encompass the entire cortex whereas PAR-2 was found in cytoplasmic puncta (Figure 2C–F and legend). This pattern is strikingly similar to that seen in partial loss of function mutants or weak RNAi of APC/C subunits *emb-27/Cdc16, mat-1/Cdc27, mat-2/Apc1, mat-3/Cdc23*, and *emb-30/Apc4 *[16]. The similarity in the range of phenotypes induced by RNAi of B0511.9 and APC/C components argues that B0511.9 functions with the APC/C.

### B0511.9 shows homology to the APC/C subunit Cdc26

There are two alternatively spliced isoforms of B0511.9 inferred from the sequence of ESTs, called B0511.9a and B0511.9b, which are predicted to encode proteins of 175aa and 187aa, respectively [27]. The final intron predicted in Wormbase [27] is not removed in the ESTs, making the proteins shorter than originally proposed due to an earlier stop codon. Homology searches using Blastp [28] with standard settings against the non-redundant protein database (NCBI) did not uncover any similarity of B0511.9 to any known protein. However, Blastp searches against the *S. cerevisiae *protein database revealed homology of the N-terminus of B0511.9 to the N-terminus of Cdc26.

**Figure 3:**
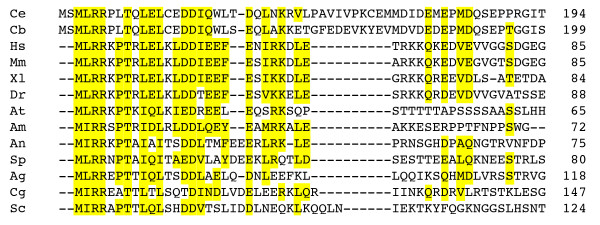
**Multiple sequence alignment of N-terminal sequences from Cdc26 orthologues**. All sequences start at the initiator methionine, and numbers give the total length of the protein. Conserved residues are highlighted in yellow. Ce, *Caenorhabditis elegans *(NP_740913, B0511.9); Cb, *Caenorhabditis briggsae *(CAE67051); Hs, *Homo sapiens *(NP_644815); Mm, *Mus musculus *(NP_647452); Xl, *Xenopus laevis *(BP677104); Dr, *Danio rerio *(NP_001004005); At, *Arabidopsis thaliana *(AAN10198); Am, *Apis mellifera *(XP_001122028); An, *Aspergillus nidulans *(AI210365); Sp, *Schizosaccharomyces pombe *(O13916, Hcn1); Ag, *Ashbya gossypii *(NP_984005); Cg, *Candida glabrata *(CAG60767); Sc, *Saccharomyces cerevisiae *(NP_116694).

Budding yeast *CDC26 *encodes a small protein of 124 aa that is dispensable for proliferation at 25°C but essential for progression through mitosis at 37°C [29]. The protein resides within the APC/C where it stabilizes the association of the TPR repeat proteins Cdc16 and Cdc27 with other subunits of the complex [30,31]. Proteins related to Cdc26 have been found in the APC/C isolated from fission yeast and human cells [32,33]. Although the B0511.9 sequence is longer than those of other Cdc26 orthologues, it shares with all these proteins a conserved pattern of charged and large, hydrophobic residues at the very N-terminus (Figure 3). In contrast, the C-terminal regions of Cdc26 orthologues lack detectable sequence conservation. Indeed, the first 71 amino acids of *S. cerevisiae *Cdc26 are sufficient for proliferation albeit at a reduced rate [29].

### B0511.9 complements a budding yeast *cdc26 *mutant

**Figure 4:**
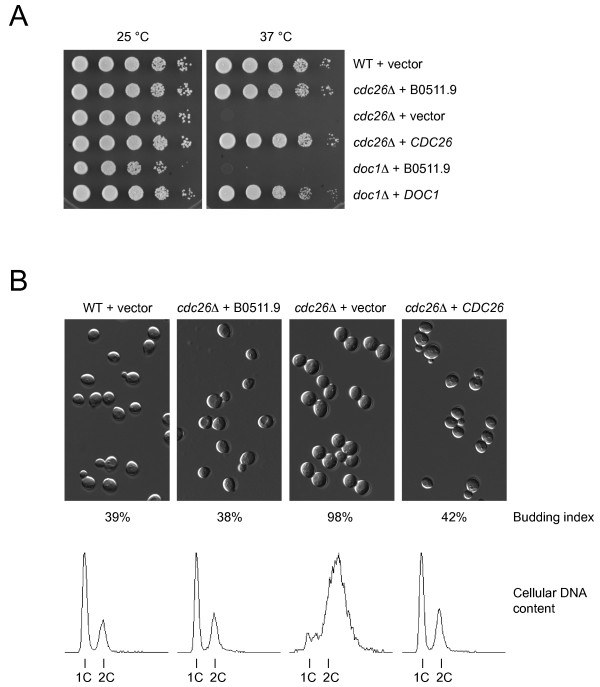
**The *C. elegans *B0511.9 gene complements the proliferation defect of yeast cells lacking the *CDC26 *gene**. (A) *S. cerevisiae *cells containing a deletion of *CDC26 *were transformed with a plasmid expressing the *C. elegans *B0511.9 gene from the yeast *PGK *promoter, or with the empty vector, or with a plasmid containing the yeast *CDC26 *gene. Wild type cells (WT) transformed with vector and cells lacking the *DOC1/APC10 *gene transformed with plasmids containing B0511.9 or the yeast *DOC1 *gene served as controls. Transformants were grown at 25°C in selective medium, normalized for cell density, and tenfold serial dilutions were spotted onto plates with rich medium. Shown are plates incubated at 25°C for 36 hours and at 37°C for 24 hours. (B) Strains from (A) were grown at 25°C and then shifted to 37°C for 5 hours. Shown are differential interference contrast pictures of the cells. Numbers indicate the percentage of budded cells in the indicated cultures. Graphs show cellular DNA content measured with a flow cytometer.

To test whether B0511.9 could functionally replace Cdc26, we expressed it in *S. cerevisiae *cells lacking the endogenous *CDC26 *gene. As shown in Figure 4A, the B0511.9 plasmid but not the empty vector restored proliferation of *cdc26Δ *mutant cells at 37°C. Complementation was remarkably robust: *cdc26Δ *cells expressing B0511.9 grew with wild-type kinetics and were normal with respect to cell size, budding index, and cellular DNA content (Figure 4B). Expression of B0511.9 failed to rescue the temperature-sensitive growth defect of cells lacking another APC/C subunit, Doc1/Apc10. This result demonstrates that B0511.9 provides Cdc26 function to the yeast cells but does not rescue a general defect in APC/C activity.

## Conclusion

Our functional and phenotypic assays indicate that B0511.9 encodes the *C. elegans *APC/C subunit Cdc26 and accordingly we have named it *cdc-26*. Inhibition of *cdc-26 *activity leads to the same range of defects as seen after inhibition of other APC/C subunits, namely embryonic polarity defects after weak knockdown, and meiotic metaphase I arrest following strong knockdown. Consistent with the role of yeast Cdc26 in stabilizing the association of Cdc16 with other subunits, a large-scale two-hybrid study in *C. elegans *showed that CDC-26 binds to APC/C component EMB-27/Cdc16 [34].

Previous studies had identified *C. elegans *homologs of 9 of the 12 known human APC/C subunits (reviewed in [18]). No sequences with significant matches to Cdc26, Apc7, or Apc13 had been found. This study illustrates the power of RNA interference screens coupled with detailed phenotypic analyses in assigning gene function. RNAi embryo videorecording data for hundreds of genes are available [4,7-9]. In many cases, the defects observed in these movies give insight into the biological process affected. Further study of genes in different phenotypic classes will provide a deeper understanding into the mechanisms of shared cell division processes. For example, careful analyses of these data may lead to the identification of *C. elegans *Apc7 and Apc13.

## Methods

### Strains

The following strains were used and cultured by standard methods [35]: wild-type Bristol N2, AZ212: *unc-119(ed3) ruIs32 [unc-119(+) pie-1::gfp:*:H2B] [36].

### RNA interference

RNAi was carried out by feeding [37] as in [38] using RNAi feeding clones from [3,5]. Sequences of the clones are available in Wormbase [27] as sjj_B0511.9 for B0511.9 and as sjj_F10B5.6 for *emb-27*. For the time course in Table 1, wild-type N2 L4 hermaphrodites were placed on RNAi feeding plates containing the same bacterial strain at 20°C for 24 hours, then moved to new RNAi feeding plates for the indicated collection times. Embryos laid on each plate were scored the next day for embryo hatching and terminal phenotype. Fertilized embryos were easily distinguished from unfertilized oocytes by their oblong rather than rounded shape and presence of an eggshell, visible in the dissecting microscope. For strong RNAi of B0511.9 in Figure 1, GFP::H2B L4 hermaphrodites were placed on feeding plates for 30 hours at 25°C and then scored by DIC and GFP microscopy; for RNAi of *emb-27*/Cdc16, RNAi feeding was for 20 hours. For weak RNAi of B0511.9 in Figure 2 and for videorecording analyses, wild-type N2 L4s were placed on feeding plates for 40 hours at 20°C, then embryos dissected and processed for antibody staining or videorecording.

### Embryo analyses

Videorecordings were done as in [9]. Antibody staining was done as in [22]. The PAR-3 antibody was raised in rat using GST-PAR-3 described in [21] and then affinity purified against the same protein after preclearing the serum of anti-GST antibodies. The PAR-2 antibody was raised in rabbit against N-terminally His-tagged full length PAR-2 and then affinity purified against the His-tagged N-terminus of PAR-2 (amino acids 1–100). Secondary antibodies were from Jackson Immunoresearch.

### Yeast experiments

The *cdc26Δ::KanMX4 *strain and the corresponding wild-type are in the BY4741 genetic background (*MATa his3Δ1 leu2Δ0 met15Δ0 ura3Δ0*) and were obtained from the European *Saccharomyces cerevisiae *Archive for Functional Analysis (EUROSCARF). The *doc1Δ::KanMX4 *deletion allele was introduced into the W303 background (*MATa ade2-1 trp1-1 can1-100 leu2-3,112 his3-11,15 ura3-1*).

To express *C. elegans *B0511.9a in yeast, a cDNA in the donor vector pDONR201 was obtained from [39] and then transferred to the expression destination vector pVV214 using GATEWAY recombinational cloning [40]. The resulting plasmid pJA189 contains an *URA3 *marker, the yeast 2-micron origin, and expresses B0511.9a from the *PGK *promoter. Translation starts at the second methionine of the original B0511.9a sequence where the homology with other Cdc26 orthologues begins (see Figure 3). To construct positive controls for the complementation of yeast mutants, yeast *CDC26 *and *DOC1 *were cloned into the vectors YCplac33 [41] and pRS426 [42], respectively. Standard protocols were used to transform yeast and to prepare growth media [43]. To determine the budding index > 200 cells were counted after brief sonication. To measure cellular DNA content cells were stained with propidium iodide and analyzed on a Becton Dickinson FACScan flow cytometer.

### Bioinformatics

Database searches were performed at the National Center for Biotechnology Information with Tblastn and Blastp [28]. Multiple sequence alignments were generated with ClustalX [44] and edited manually.

## Authors' contributions

YD participated in the design of the study, performed the experiments described in Figures 1 and 2 and Table 1, and helped to draft the paper; BH did the alignment in Figure 3; AB and WZ did the experiment described in Figure 4; JA conceived the study and participated in its design; JA and WZ wrote the paper. All authors read and approved the final manuscript.
